# Effect on HIV-1 Gene Expression, Tat-Vpr Interaction and Cell Apoptosis by Natural Variants of HIV-1 Tat Exon 1 and Vpr from Northern India

**DOI:** 10.1371/journal.pone.0082128

**Published:** 2013-12-19

**Authors:** Sneh Lata, Larance Ronsard, Vikas Sood, Sajad A. Dar, Vishnampettai G. Ramachandran, Shukla Das, Akhil C. Banerjea

**Affiliations:** 1 Department of Microbiology, University College of Medical Sciences and Guru Teg Bahadur Hospital, Delhi, India; 2 Laboratory of Virology, National Institute of Immunology, New Delhi, India; University of Nebraska Medical Center, United States of America

## Abstract

**Background:**

Since HIV-1 Tat and Vpr genes are involved in promoter transactivation, apoptosis, etc, we carried out studies to find out nature and extent of natural variation in the two genes from seropositive patients from Northern India and determined their functional implications.

**Methods:**

HIV-1 *tat* exon 1 and *vpr* were amplified from the genomic DNA isolated from the blood of HIV-1 infected individuals using specific primers by Polymerase Chain reaction (PCR) and subjected to extensive genetic analysis (CLUSTAL W, Simplot etc). Their expression was monitored by generating myc fusion clones. Tat exon 1 and Vpr variants were co-transfected with the reporter gene construct (LTR-luc) and their transactivation potential was monitored by measuring luciferase activity. Apoptosis and cell cycle analysis was done by Propidium Iodide (PI) staining followed by FACS.

**Results:**

Exon 1 of *tat* was amplified from 21 samples and *vpr* was amplified from 16 samples. One of the Tat exon 1 variants showed phylogenetic relatedness to subtype B & C and turned out to be a unique recombinant. Two of the Vpr variants were B/C/D recombinants. These natural variations were found to have no impact on the stability of Tat and Vpr. These variants differed in their ability to transactivate B LTR and C LTR promoters. B/C recombinant Tat showed better co-operative interaction with Vpr. B/C/D recombination in Vpr was found to have no effect on its co-operativity with Tat. Recombinant Tat (B/C) induced more apoptosis than wild type B and C Tat. The B/C/D recombination in Vpr did not affect its G2 arrest induction potential but reduced its apoptosis induction ability.

**Conclusions:**

Extensive sequence and region-specific variations were observed in Tat and Vpr genes from HIV-1 infected individuals from Northern India. These variations have functional implications & therefore important for the pathogenicity of virus.

## Introduction

Human Immunodeficiency Virus (HIV) was discovered in 1983. It has become one of the biggest health problems throughout the world despite widespread use of ARV (Anti-retroviral) agents. UNAIDS global AIDS epidemic report 2012 documented a 50 percent drop in new HIV infections; yet globally there were 34.2 million HIV-infected people at the end of 2011. The slight increase from 33.5 million in 2010 is due to the combined effect of continuing new infections, an increase (by 63%) in number of infected ARV recipients and fewer deaths (24% less from 2005) from AIDS globally. HIV-1 belongs to *Retroviridae* family. HIV-1 isolates from all over the world have been divided into four groups, namely M, N, O and P. The ‘M’ group is by far the most common type of HIV. More than 90 percent of HIV/AIDS cases are due to this group. The M group of viruses consists of at least nine pure subtypes and numerous circulating recombinant forms [1], [2], [3] and unique recombinant forms [4], [5], [6]. At least 10 percent of circulating HIV-1 strains comprise of intersubtype recombinants [7], [8], [9]. Recent studies indicate that subtype C (responsible for majority of the infections world wide (more than 56%) accounts for more than 98 percent of the infections in the Indian subcontinent [10]. The group P was recently discovered in wild gorillas. The virus had been isolated from a Cameroonian woman [11]

HIV-1 trans-activator of transcription (Tat) protein has been a key focus of HIV research since its discovery in 1985 [12] because of its crucial role in activating viral gene transcription and several other functions having significant implications for pathogenesis. Tat is a small protein (9–11 kDa) consisting of 86 to 101 amino acids depending on the subtype [13]. Tat has two exons; first exon encoding 72 amino acids is sufficient for HIV-1 LTR transactivation [14], [15], [16]. The Tat sequence has been subdivided into several distinct regions on the basis of its amino acid composition: N-terminal acidic region (aa 1–22), a cysteine-rich domain (aa 22–37), a core region (aa 38–47), a basic region (aa 48–57) and a glutamine-rich region (aa 60–76) [17]. The acidic region is known to function as activation domain [18]. The cysteine-rich domain is believed to be involved in zinc ion - mediated dimer formation [19]. The core, basic and glutamine rich regions are all involved in RNA binding and basic region also acts as a nuclear localization signal [20], [21], [22], [23], [24], [25], [26]. The C-terminal domain of Tat has been implicated in stimulating the co-transcriptional capping of HIV-1 mRNA through a direct interaction with the capping enzyme MceI [27]. The HIV-1 Tat has also been reported to have a dual role in regulating apoptosis of host cells. Exogenous Tat induces apoptosis in normal cells while it protects the HIV- 1 infected cells from apoptosis that may be advantageous for the survival of HIV-1 infected cells in vivo [28]. The HIV-1 Tat also increases NFκB mediated IL-8 secretion in T cell lines [29]. Tat has also been reported to result in upregulation of CCR5 and downregulation of CXCR4 [30].

HIV-1 Vpr is a 14-kDa, 96-amino-acid protein. Vpr has three alpha helices. These helices are connected by loops and are folded around a hydrophobic core [31] surrounded by a flexible N-terminal domain and a C-terminal arginine-rich region that are negatively and positively charged, respectively. Vpr is packaged in significant quantities into viral particles [32]. Vpr helps in the active nuclear translocation of the HIV-1 pre-integration complex (PIC) in nondividing cells by interacting with the nuclear transport pathway [33], [34], [35], [36]. Vpr induces arrest at the G_2_/M phase of the cell cycle [37], [38]. Vpr up-regulates HIV replication as a result of its cell cycle-modulating activity [39], [40]. Vpr also induces apoptosis of infected cells [41].

Several vaccines using HIV-1 accessory proteins like Tat toxoid had been developed by different researchers [42], [43], [44] but they were not successful because of high genetic variability of HIV, high error rate of reverse transcriptase [45], [46], [47], fast turnover of virions in HIV infected individuals [48] and homologous recombination [7], [8]. So, it becomes impossible for the immune system to keep up with the antigenic mosaic of the pathogen. Thus, the study of natural variations in HIV-1 proteins occurring in a population will be helpful in designing vaccines. The genetic variations in HIV-1 Tat exon 1 have been reported from different regions of India [49], [50] but their functional implications have not been investigated. Tat and Vpr proteins have been found to synergistically enhance the transcriptional activity of HIV-1 LTR by structural and functional interaction with each other [51]. The aim of the present study was to find out the nature of genetic variations in Tat exon 1 and Vpr found in HIV-1 infected individuals from North India and to determine their functional significance. We are reporting some interesting and unique genetic variants of Tat exon 1 and Vpr including intersubtype recombinants from North India.

## Materials and Methods

### HIV-1 infected patients studied

Twenty four HIV-1-infected individuals, some on anti-retroviral treatment (ART) and some naïve, were randomly selected for this study from the Delhi/Uttar Pradesh region of North India. They were registered at the ART Clinic of the Guru Teg Bahadur Hospital, Delhi. The clinical profile of these individuals is given in Table 1. Peripheral blood samples (2 ml) were collected in EDTA (Ethylene diamine tetra-acetic acid) containing vials from the selected individuals.

**Table 1:** Clinical profile of HIV-1 infected individuals studied.

S. No.		ART	Non ART
1	No. of patients studied	13	11
2	Age (in yrs)	14–52	19–50
3	Sex (M/F/TG)	7/6/0	6/4/1
4	Mode of Transmission (H/MC/BT/UN)	9/1/1/2	8/0/0/3
5	CD4 count	<400	≤200
6	Co-infection (Tuberculosis)	5	-
7	WHO Clinical Stage (I/II/III/IV)	1/5/5/2	1/-/-/-

**Abbreviations:** M – Male, F – Female, TG – Transgender, H – Heterosexual, MC – Mother to Child, BT – Blood Transfusion, UN – Unknown.

### Ethics Statement

Blood samples were collected from the HIV-1 infected patients after getting there written consent. This study was approved by the Research Project Advisory Committee, Institutional Biosafety Committee and Institutional Ethical Committee – Human research of University College of Medical Sciences and Guru Teg Bahadur Hospital, Delhi, India. This is mentored by National AIDS Control Organization which is a wing of Ministry of health and Family welfare, Government of India that provides free ART to HIV seropositive patients under a structured HIV/AIDS Control Programme.

### Genomic DNA Isolation and Polymerase Chain Reaction

The genomic DNA was isolated from the peripheral blood collected from the selected HIV-1 infected individuals using HiPurA™ Blood Genomic DNA Miniprep Spin Kit (HiMedia). The HIV-1 *tat* exon 1 and Vpr were amplified from these DNA samples by PCR (Polymerase Chain Reaction) with Taq DNA Polymerase (Takara) using specific primers. The primers used for amplification of *tat* exon 1 were FP, SalI, 5′-GAGGTCGACCATGGAGCCAAGTAGATC-3′ and RP, NotI, 5′- GAGGCGGCCGCCTATTGCTTTGATATAAGA-3′. Primers used for amplification of *vpr* were FP, BglII, 5′-GGCAGATCTCTATGGAACAAGCCCCAGAAGAC-3′ and RP, NotI, 5′- GGCGCGGCCGCCTAGGATCTACTGGCTCCATTTCTT - 3′. The cycling conditions used for the PCR reactions were: initial denaturation at 95°C for 5 minutes, cyclic denaturation at 95°C for 30 seconds, primers annealing at 68°C for *tat* exon 1 and 63°C for *vpr* for 30 seconds, cyclic extension at 72°C for 30 seconds, final extension at 72°C for 7 minutes and 30 cycles. The PCR amplicons of *tat* exon 1 and *vpr* were run on 1.5 percent agarose gel and bands were purified with QIAquick Gel Extraction Kit (Qiagen).

### Cloning & expression vectors

The purified PCR amplicons were initially cloned into pGEM-T Easy vector (Promega) by TA cloning. For mammalian expression of unique *tat* exon 1 and *vpr* variants, they were cloned in pCMV-Myc vector (Clontech). Their pGEM-T easy clones as well as empty pCMV-Myc vector were digested with respective restriction enzymes, run on 1.5 percent agarose gel, purified and ligated together using T4 ligation kit (New England Biolabs). DH5α strain of E.Coli was used for cloning experiments. pCMV-Myc-B Tat, pCMV-Myc-C Tat, pCMV-Myc-B Vpr, pCMV-Myc-C Vpr, pBlue3′LTR-luc, pBlue3′LTR-luc-C (NIH AIDS Reagent Programme) plasmid constructs were used in different experiments.

### Genetic analysis of Tat exon 1 and Vpr variants

The pGEM-T Easy clones of *tat* exon 1 and *vpr* variants were subjected to sequencing in both directions using T7 and SP6 specific primers. These sequences were then aligned against HIV-1 reference sequences downloaded from HIV database (http://www.hiv.lanl.gov) using Clustal W 2.1 [52]. Sequences were also analyzed by Sim Plot version 3.5.1 for recombination analysis.

### HIV-1 classification by phylogenetic analysis and dN/dS ratio

The nucleotide sequences were assembled and error checked by using BLAST program (NCBI, USA) to rule out the potential laboratory errors. These sequences were aligned with consensus sequences of HIV-1 strains of all subtypes using the ClustalW 2.1. The phylogenetic trees were constructed using neighbour joining method with kimura two-parameter distance matrix in MEGA5 software. The reliability of node was tested using the bootstrap method with 500 replicates for both Tat exon-1 and Vpr variants. SNAP v1.1.0 tool [53] was used to determine the evolutionary selection pressure in our variants.

### Cell culture and transfection

HEK-293T (Human Embryonic Kidney 293 cells) and Hela (Human Cervical Cancer Cell line) cells (NIH AIDS Reagent Programme) were maintained in Dulbecco's modified Eagle's medium (Himedia) with heat inactivated 10 percent fetal bovine serum (Biological Industries) and 100 units penicillin, 0.1 mg streptomycin and 0.25 µg amphotericin B per ml at 37°C in the presence of 5 percent CO_2_. All transfections were performed using Lipofectamine 2000 (Invitrogen) reagent.

### Immunoblot Analysis

HEK-293 T cells were grown to 80 percent confluency in a six-well plate and were transfected with plasmid expression vectors encoding the desired proteins. After 24 hrs of transfection, cells were harvested and lysed with cell lysis buffer (1% NP-40, 50 mM TrisCl, pH 8, 300 mM NaCl, 5 mM EDTA, 15 mM MgCl_2_, 2 mM DTT). Protein concentration was quantitated using BCA Protein Assay kit (Pierce, Thermo Scientific). Equal amounts of protein were resolved by 12 percent SDS PAGE (Sodium dodecyl sulfate poly-acrylamide gel electrophoresis) and were transferred to nitrocellulose membrane (mdi). The membranes were blocked with 1 percent BSA (Bovine Serum Albumin) (Sigma) and 5 percent non fat dry milk (Himedia) in PBS (Phosphate Buffer Saline) and washed thrice with PBS containing 1% Tween 20 (MERCK). Primary antibodies used were anti-myc (Clontech) and anti-GAPDH (Cell Signalling). The horseradish peroxide (HRP)-conjugated anti-mouse and anti-rabbit secondary antibodies were used. Blots were developed using ECL (Enhanced Chemiluminiscent) reagent.

### Cycloheximide Chase assay

To compare the stability of natural variants of Tat exon 1 and Vpr with that of wild type, cycloheximide (CHX) chase was performed. HEK-293T cells were transfected with 2 µg each of respective plasmid. After 24 hrs of transfection, cells were treated with cycloheximide (100 µg/ml) and cell lysates were prepared after 0 hr, 2 hrs, 4 hrs, 6 hrs and 8 hrs of CHX treatment. Cell lysates were resolved by 12 percent SDS PAGE followed by immunoblotting as described above.

### Luciferase Reporter Assay

HIV-1 LTR transactivation was measured by Dual Luciferase Reporter (DLR) assay kit (Promega). 293 T cells were co-transfected with HIV-1 LTR luciferase reporter construct, plasmids encoding wild type Tat and Vpr proteins and variant Tat exon 1 and Vpr constructs. To measure the co-activation of HIV-1 LTR by Tat exon 1 variants and B Vpr or Vpr variants and B Tat, 293T cells were transfected with HIV-1 LTR luciferase reporter construct along with B Tat, B Vpr and variant Tat exon 1 and Vpr constructs alone or with wild type B Vpr and B Tat respectively. Renilla luciferase construct was used as a control to normalize the transfection efficiency. Empty pcDNA3.1 vector was added to equalize the amount of DNA transfected in each well. Twenty-four hours post-transfection, cells were lysed in passive lysis buffer (Promega). Luciferase activity was measured by luminometer using two substrates (Promega), one for firefly luciferase and second for renilla luciferase (mixed with stop and glo buffer) by luminometer (Tecan). The firefly luciferase activity was divided by renilla luciferase acivity to give the true reporter luciferase activity.

### Flow Cytometry

For apoptosis analysis, Hela cells were transfected with expression plasmids encoding the desired proteins for 24 hrs. For cell cycle analysis, cells were also treated with 40 µM Z VAD-FMK [54] (Pan caspase inhibitor). The cells were harvested using Trypsin EDTA and washed twice with cold PBS. Cells were fixed and permeabilized in cold 70 percent ethanol on ice for 30 minutes. Cells were then treated with RNase A for 1 hr at room temperature. After 1 hr, cells were stained with propidium iodide, PI (50 µg/ml) and analyzed by flow cytometry. FACS data were analyzed using WINMDI version 2.9.

### Statistical analysis

Statistical calculations and analyses were performed in Graph Pad Prism (Version 5.00). The P values less than 0.05 were only considered statistically significant.

## Results

### Description of Selected HIV-1 infected individuals

A total of twenty four HIV-1 infected patients were selected from Delhi/Uttar Pradesh regions of North India for this study. Their clinical history is summarized in Table 1. Thirteen patients were receiving ART treatment and others were not. Most of them acquired HIV infection through heterosexual transmission. Their CD4 counts were below 400 at the time of sample collection. Five of the ART treated patients were co-infected with *Mycobacterium tuberculosis*.

### Multiple Sequence Analysis

**Figure 1:**
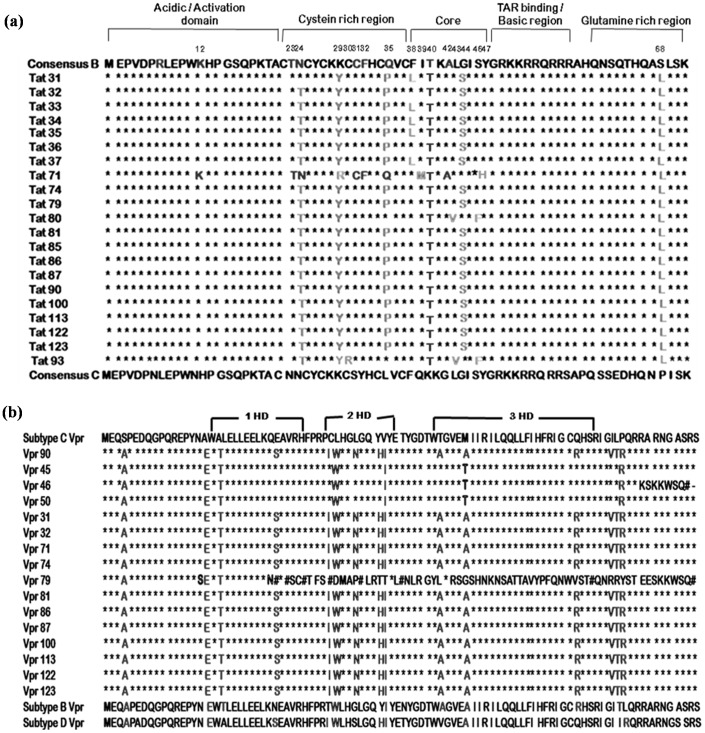
Amino acid sequence alignment of Tat exon 1 and Vpr sequences of HIV-1 infected patients. (**a**) Tat exon 1 sequences were aligned against HIV-1 subtype B and C consensus sequences by Clustal W. In the sequence alignment, amino acids identical to Consensus C are denoted by stars (*), amino acids identical to Consensus B, mutations conserved in all test sequences and unique mutations are shown alphabetically. The positions of the mutated amino acids are indicated above the alignment. (**b**) Amino acid sequences of Vpr variants from HIV-1 infected patients were aligned against HIV-1 subtype B, C and D reference sequences by Clustal W. In the sequence alignment, amino acids identical to Consensus C are denoted by stars (*). Amino acids identical to Consensus B & D, mutations common in test sequences and unique mutations are shown in alphabets.

Amino acid sequences of Tat exon 1 from HIV-1 infected samples were aligned against HIV-1 subtype B and C consensus sequences [Fig. 1(a)]. Some of the mutations (N24T, K29Y, L35P, G44S and P68L) were conserved in most of the sequences. Five sequences (Tat 31, 33, 34, 35 & 37) had F38L mutation in core region. One sequence (Tat 71) resembled B Tat in amino-terminal region with three point mutations (K29R in cystein rich domain and I39M and Y47H in core region) and C Tat in carboxy-terminal region. Two sequences (Tat 80 and Tat 93) had L43V and S46F mutations. The C30R mutation was found in only one sequence (Tat 93). Amino acid sequences of Vpr from HIV-1 infected samples were aligned against HIV-1 subtype B, C and D consensus sequences [Fig. 1(b)]. Three sequences Vpr 45, Vpr 46 and Vpr 50 were found to be mosaic of subtype B, C and D with one point mutation, M59T. The sequence of Vpr 45 and Vpr 50 was same. In C terminal region of Vpr 46 sequence, there was a frameshift mutation resulting in change of amino acid sequence and formation of premature stop codon at the C-terminal end of protein. Vpr 79 was showing premature truncation. It was due to the insertion of four nucleotides in N terminal region resulting in the formation of premature stop codon and entirely different amino acid sequence onwards.

### Phylogenetic analysis

**Figure 2:**
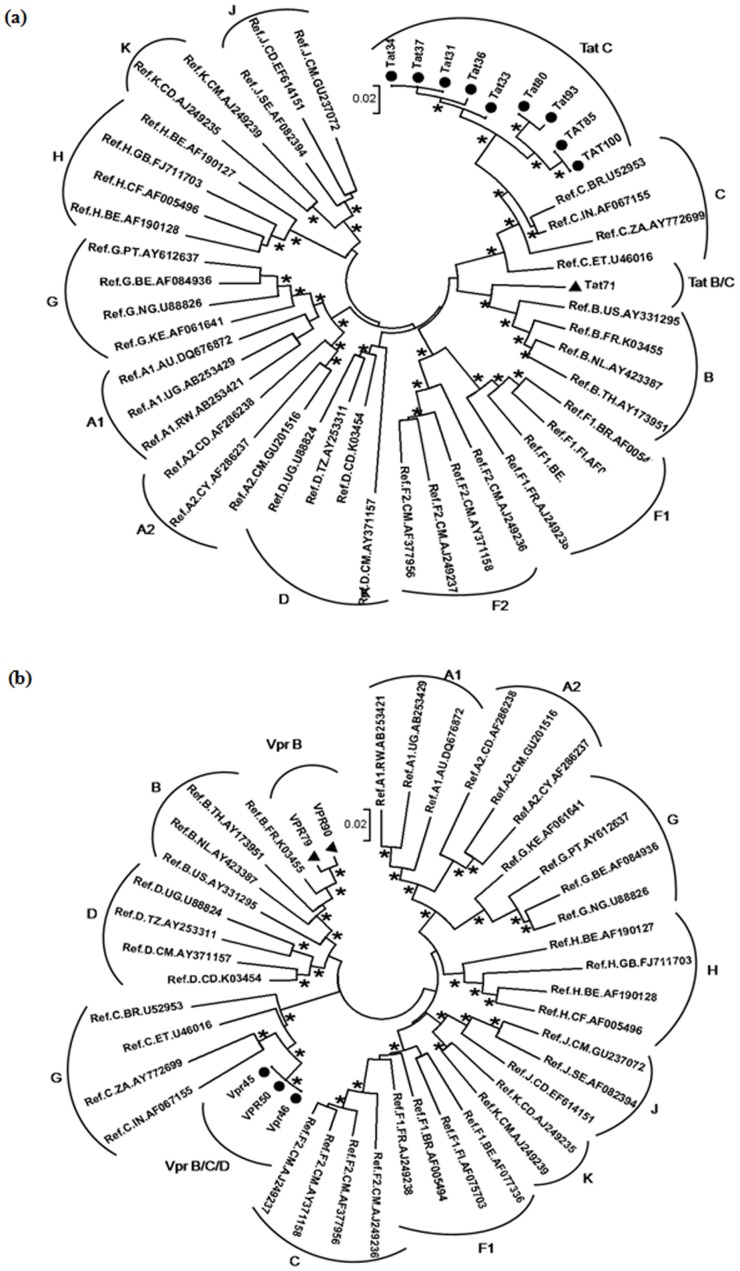
HIV-1 subtyping based on global subtype references. Phylogenetic analysis was performed for Tat exon-1 and Vpr variants with M (A to K including A1, A2, F1, and F2) group. Each reference sequence was labelled with subtype, followed by country of isolation and accession number. The bootstrap probability (>65%, 1,000 replicates) was indicated with asterisk (*) at the corresponding nodes of the tree and the scale bar represents the evolutionary distance of 0.02 nucleotides per position in the sequence. (**a**) It represents the phylogenetic tree of Tat exon-1 variants with M group. The filled circles represent C variants and the filled triangles represent recombinants. (**b**) It represents the phylogenetic tree of Vpr variants with M group. The filled triangles represent B variants and the filled circles represent recombinants.

The nucleotide sequences of Tat exon-1 and Vpr were aligned by ClustalW 2.1 [52] and analyzed for subtypes by phylogenetic analyses using neighbour joining method [55] with Kimura two parameter distance matrix in MEGA5 software [56] with M group subtypes (A-K) and subsubtypes (A1, A2, F1 and F2) which showed most of our Tat exon-1 variants clustered with subtype C and certain variants clustered in between subtypes B and C indicating the presence of B/C recombination [Figure 2(a)]. Similar analyses for Vpr variants showed most of our variants branched with subtype B and certain variants clustered with subtype C [Figure 2(b)].

### Evolutionary selection based on dN/dS ratio

The mode of selection pressure that occurred at Tat exon-1 and Vpr variants was calculated by taking the average dN/dS values within the predicted subtype using SNAP v1.1.0 (Synonymous Non-synonymous Analysis Program). SNAP calculates non-synonymous (dN) and synonymous (dS) substitution rates based on a set of codon-aligned nucleotide sequences (dN/dS ratio) [57]. The dN/dS value for Tat exon-1 and Vpr variants were 0.4 to 0.9 (Table 2) and 0.1 to 0.7 (Table 3)fold respectively, e.i dN/dS value less than one denote the purifying selection and the value greater than one denote the positive selection. Both our Tat exon-1 and Vpr variants including B/C and B/C/D recombinants showed less than one value indicating the purifying selection among our North Indian population. Further, the type of selection that occurred between B and C variants was calculated which revealed that the average divergence at B and C isolates were less than one value implying purifying selection (Table 2 and 3).

**Table 2:** dN/dS calculation for Tat exon-1 variants.

Samples	dN/dS (Consensus C)	dN/dS (Consensus B)	Predicted Subtypes	Evolutionary Selection
Tat31	0.5318	0.6874	C	Purification
Tat 32	0.5195	0.6459	C	Purification
Tat33	0.5318	0.6874	C	Purification
Tat 34	0.5318	0.6874	C	Purification
Tat 35	0.5318	0.6874	C	Purification
Tat 36	0.5195	0.6459	C	Purification
Tat 37	0.5318	0.6874	C	Purification
Tat80	0.5586	0.7047	C	Purification
Tat93	0.4785	0.6234	C	Purification
Tat71	0.5486	0.9947	B/C	Purification
Tat 74	0.5526	0.6858	C	Purification
Tat 79	0.5526	0.6858	C	Purification
Tat 81	0.5526	0.6858	C	Purification
Tat 85	0.5195	0.6459	C	Purification
Tat 86	0.5526	0.6858	C	Purification
Tat 87	0.5526	0.6858	C	Purification
Tat 90	0.5526	0.6858	C	Purification
Tat 100	0.5195	0.6459	C	Purification
Tat 113	0.5526	0.6858	C	Purification
Tat 122	0.5526	0.6858	C	Purification
Tat 123	0.5526	0.6858	C	Purification

**Table 3:** dN/dS calculation for Vpr variants.

Samples	dN/dS (Consensus C)	dN/dS (Consensus B)	Predicted Subtypes	Evolutionary Selection
Vpr 31	0.1326	0.3289	B	Purification
Vpr 32	0.1326	0.3289	B	Purification
Vpr 71	0.1326	0.3289	B	Purification
Vpr 74	0.1326	0.3289	B	Purification
Vpr79	0.7392	0.9519	B	Purification
Vpr 81	0.1326	0.3289	B	Purification
Vpr 86	0.1326	0.3289	B	Purification
Vpr 87	0.1454	0.3979	B	Purification
Vpr90	0.1241	0.2143	B	Purification
Vpr 100	0.1326	0.3289	B	Purification
Vpr 113	0.1326	0.3289	B	Purification
Vpr 122	0.1441	0.3956	B	Purification
Vpr 123	0.1326	0.3289	B	Purification
Vpr45	0.1763	0.1122	B/C/D	Purification
Vpr46	0.7005	0.2154	B/C/D	Purification
Vpr 50	0.1763	0.1122	B/C/D	Purification

### Sim Plot analysis

**Figure 3:**
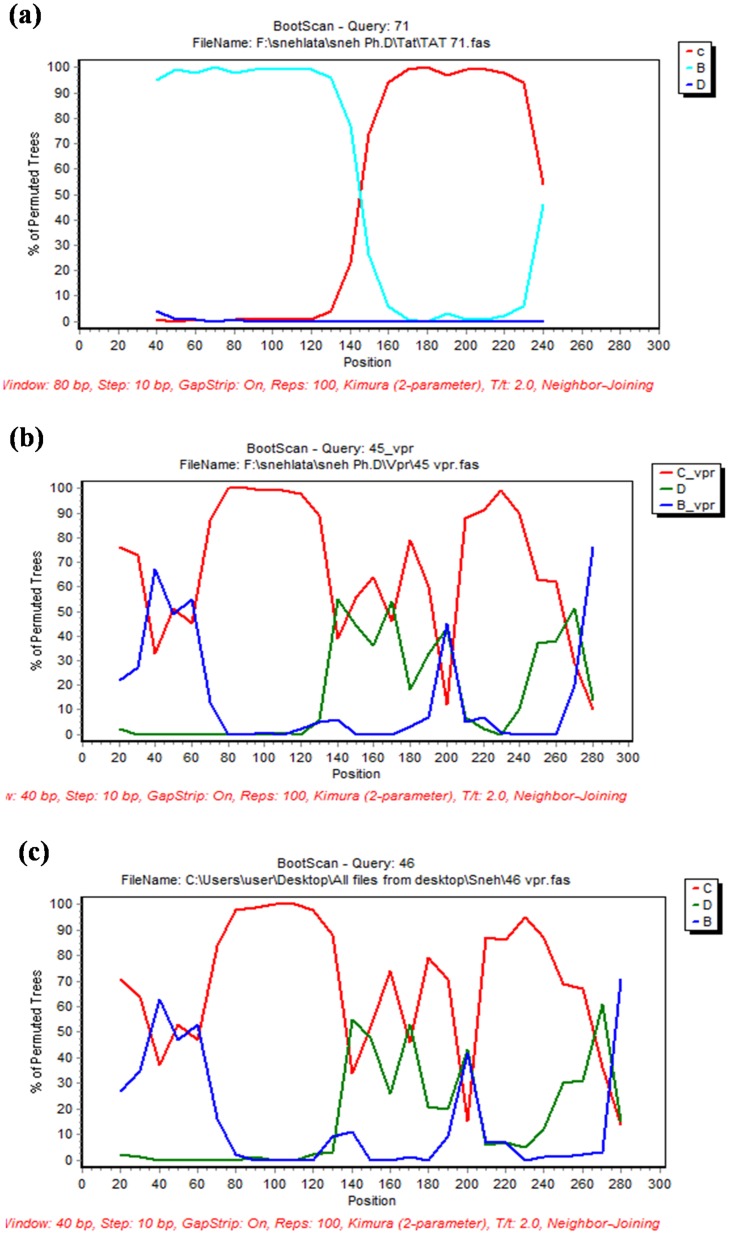
Recombination analysis of Tat exon 1 and Vpr variants. Sequences of Tat 71, Vpr 45 and Vpr 46 were analyzed by Sim Plot. They were aligned with HIV-1 subtype B, C and D consensus sequences and subjected to the boot scan analysis. Tat 71 was turned out to be a recombinant of subtype B and C. Vpr 45 and Vpr 46 were found to be recombinant of subtype B, C and D. (**a**) Boot scan analysis of Tat 71 (**b**) Boot scan analysis of Vpr 45 (**c**) Boot scan analysis of Vpr 46.

To check the presence of recombination, the sequences of Tat exon 1 and Vpr variants were analyzed by Sim Plot. The nucleic acid sequence of variants were aligned with reference sequences and subjected to boot scan analysis using Sim Plot version 3.5.1. Boot scan analysis of Tat 71 showed the presence of B/C recombination where N-terminal region was derived from subtype B but it was identical to subtype C towards C-terminal half [Fig. 3 (a)]. Other Tat exon 1 variants showed complete similarity to subtype C when subjected to similar analysis [Fig S1]. Boot scan analysis of Vpr 45 or Vpr 50 (as both sequences are same) [Fig. 3 (b)] and Vpr 46 [Fig. 3 (c)] showed B/C/D recombination in their sequences.

### Comparison of expression & stability of Tat exon 1 and Vpr variants

**Figure 4:**
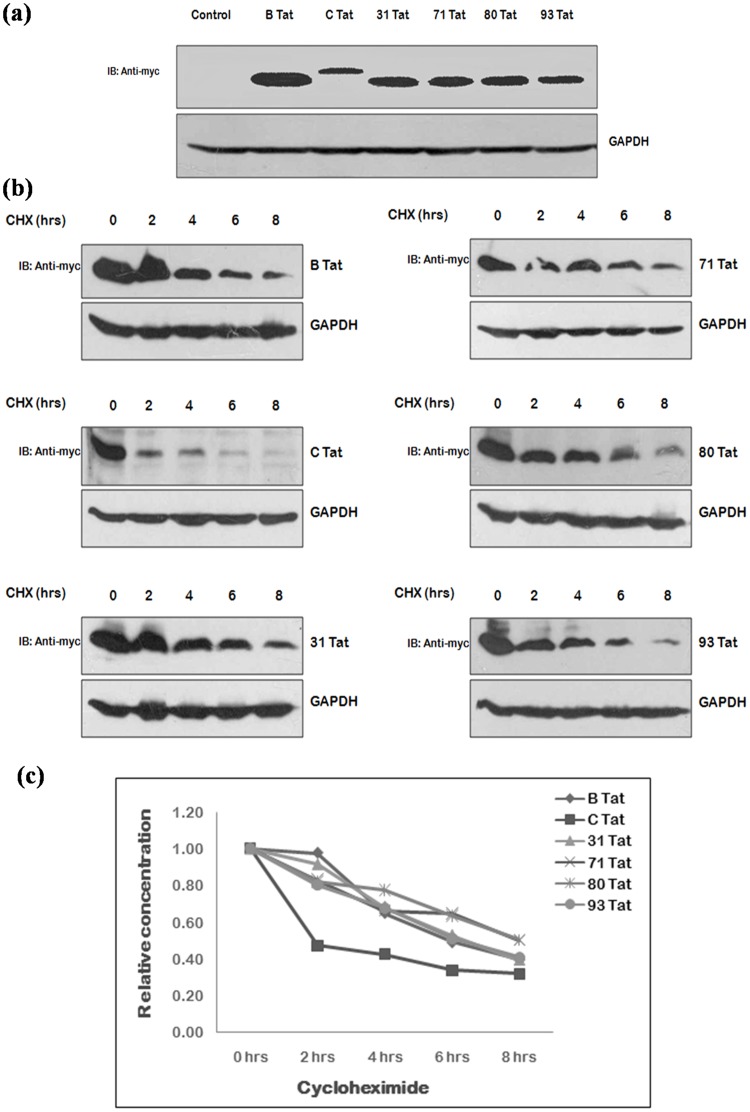
Expression and stability of Tat exon 1 variants. (**a**) 293 T cells were transfected with myc fusion constructs of wild type B Tat, C Tat and Tat exon 1 variants (2 µg each) using Lipofectamine 2000; the cell lysates were prepared 24 hrs post-transfection and were run on 12 percent SDS PAGE. Western blotting was done using c-myc monoclonal mouse antibody as primary antibody and HRP labeled anti-mouse antibody as secondary antibody. GAPDH was probed as a loading control. (**b**) 293T cells were transfected with 2 µg each of myc fusion clones of wild type subtype B Tat, C Tat and Tat exon 1 variants and after 24 hrs of transfection, cells were treated with cycloheximide, CHX (100 µg/ml) and harvested at the intervals of 2 hrs upto 8 hrs. Cell lysates were run on 12 percent SDS PAGE and blots were probed with c-myc monoclonal antibody and GAPDH antibody (loading control) (**c**) The relative concentration of protein at different time points was measured was measuring the density of the band in the each case and were line plotted against the duration of CHX treatment.

**Figure 5:**
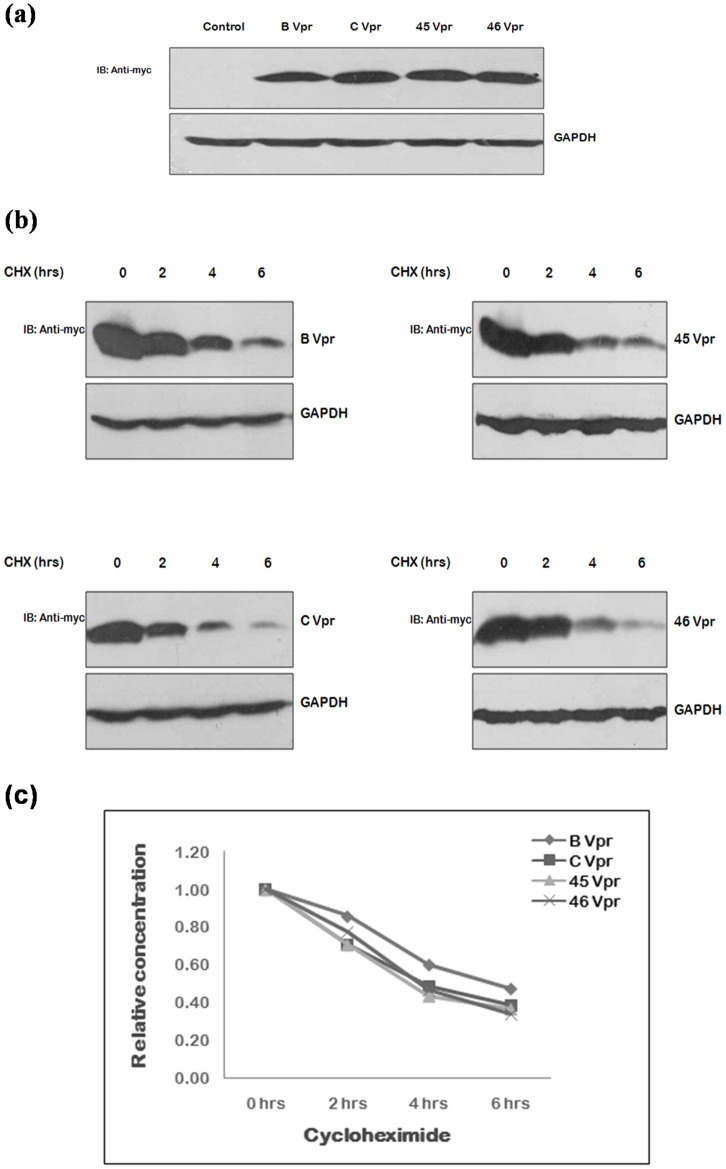
Expression and stability of Vpr natural variants. (**a**) 293 T cells were transfected with myc fusion constructs of wild type B Vpr, C Vpr and Vpr variants (2 µg each) using Lipofectamine 2000; the cell lysates were prepared 24 hrs post-transfection and were run on 12 percent SDS PAGE. Western blotting was done using c-myc monoclonal mouse antibody as primary antibody and HRP labeled anti-mouse antibody as secondary antibody. GAPDH was probed as a loading control. (**b**) 293T cells were transfected with 2 µg each of myc fusion clones of wild type subtype B Vpr, C Vpr and Vpr variants and after 24 hrs of transfection, cells were treated with cycloheximide, CHX (100 µg/ml) and harvested at the intervals of 2 hrs upto 6 hrs. Cell lysates were resolved by 12 percent SDS PAGE and blots were probed with c-myc monoclonal antibody and GAPDH antibody (loading control) (**c**) The relative concentration of protein at different time points was measured was measuring the density of the band in the each case and were line plotted against the duration of CHX treatment.

Of the natural variants found, the interesting and unique variants of Tat exon 1 i.e. Tat 31, Tat 71, Tat 80 & Tat 93 and variants of Vpr i.e. Vpr 45 and Vpr 46 were selected for functional characterization. The sequence of Vpr 79 was showing the premature stop codon formation, so no functional studies were performed with this sample further. The effect of mutations found naturally in Tat exon 1 and Vpr on their stability, if any, was investigated by cycloheximide chase assay using their myc fusion clones followed by immunoblotting. Mutant clones were transfected in 293T cells using wild type proteins as controls. After 24 hrs, cells were treated with cycloheximide and cell lysates were prepared after every 2 hrs. Although the expression of all Tat exon 1 variants was found to be less than that of B Tat and more than C Tat [Fig. 4(a)], their half lives were almost similar to that of B Tat [Fig. 4(b,c)]. The expression and stability of Vpr variants was found to be similar to that of wild type [Fig. 5].

### HIV-1 LTR transactivation potential of Tat exon 1 and Vpr variants

**Figure 6:**
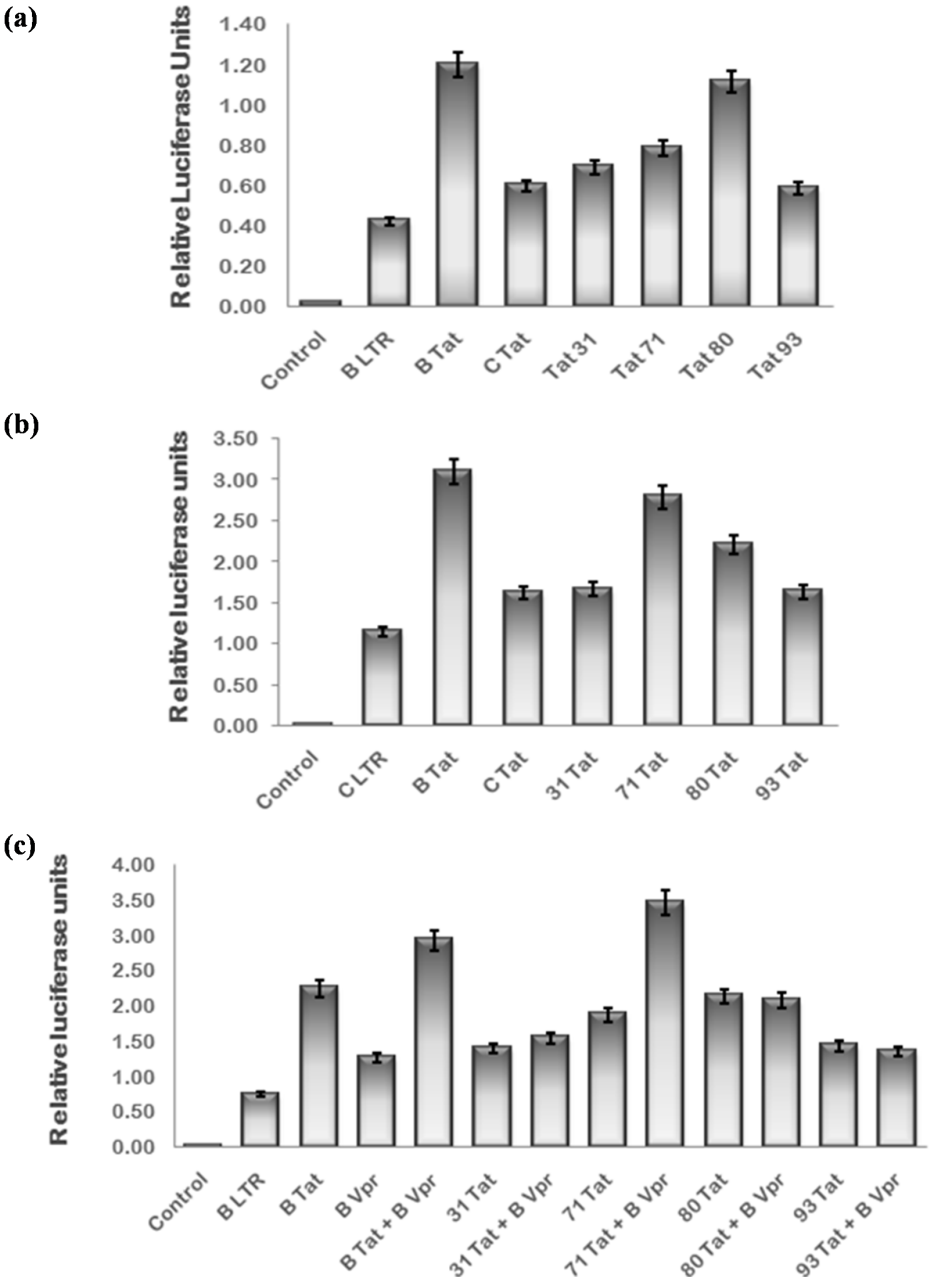
HIV-1 LTR activation potential of natural variants of Tat exon 1. 293 T cells were transfected with HIV-1 subtype B LTR or C LTR luciferase reporter plasmid (50 ng), renilla luciferase as normalization control and B Tat, C Tat & variant Tat exon 1 expression plasmids (100 ng) alone or in combination with B Vpr using Lipofectamine 2000. pcDNA 3.1 was transfected to equalize the amount of DNA transfected in each well. After 24 hrs, cells were harvested and lysed in passive lysis buffer. Luciferase activity was measured by luminometer. (**a**) HIV-1 B LTR activation by Tat 80 was comparable to that of B Tat and that by Tat 71 was less than B Tat. B LTR actvation by other variants was comparable to C Tat. (**b**) HIV-1 C LTR activation by Tat 71 was more than C Tat and comparable to B Tat. C LTR actvation by other variants was almost similar to C Tat. (**c**) The co-opeartive transactivation of B LTR by Tat 71 with B Vpr was more than B Tat while other variants did not show co-operative interaction with B Vpr as expected. The data shown here represents the mean value of at least three separate transfection experiments. The p value was less than 0.05 for each sample.

**Figure 7:**
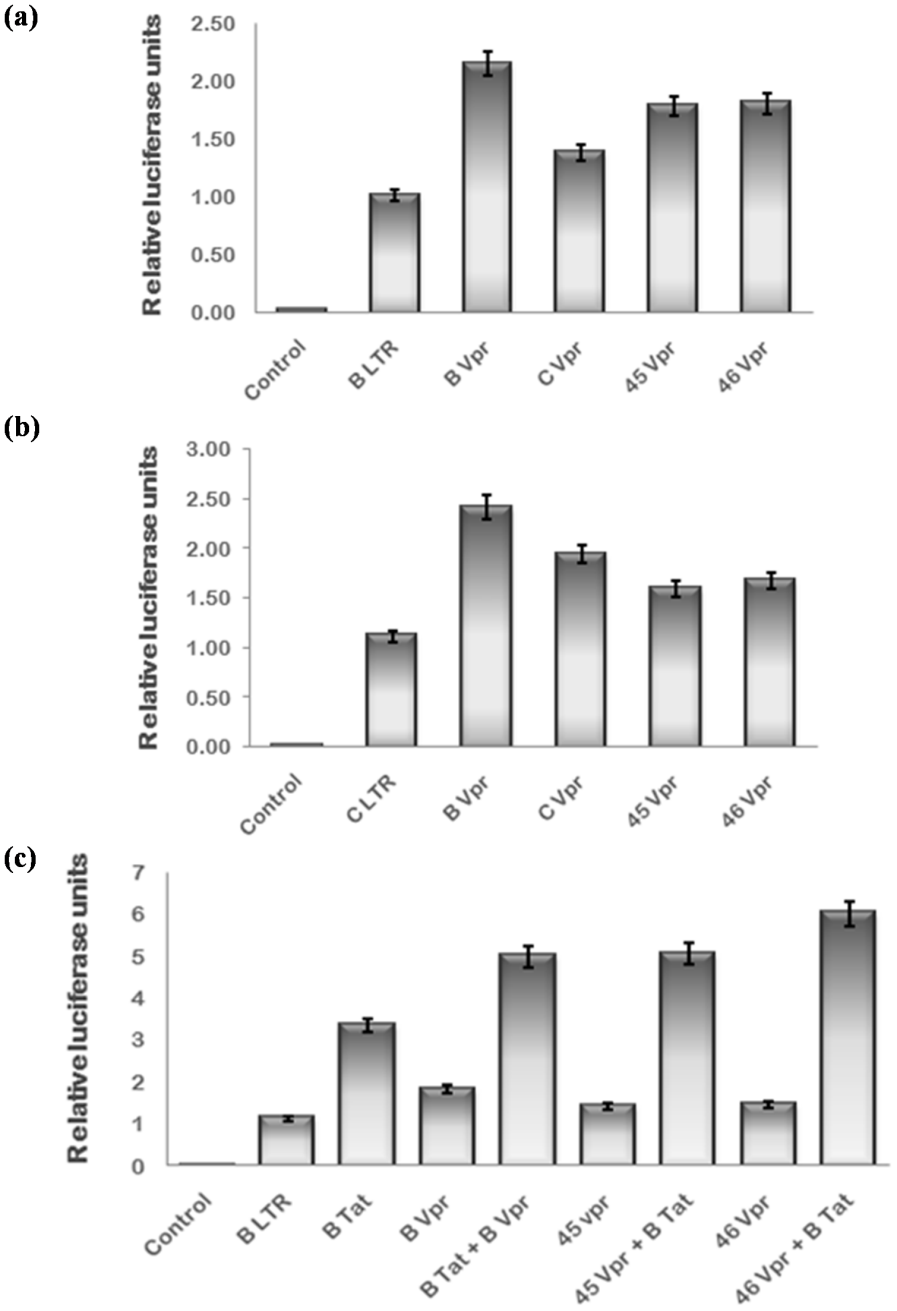
HIV-1 LTR activation potential of natural variants of Vpr. HEK-293 T cells were transfected with HIV-1 subtype B LTR or C LTR luciferase reporter plasmid (50 ng), renilla luciferase as normalization control and B Vpr, C Vpr & variant Vpr expression plasmids (100 ng) alone or in combination with B Tat using Lipofectamine 2000. pcDNA 3.1 was transfected to equalize the amount of DNA transfected in each well. After 24 hrs, cells were harvested and lysed in passive lysis buffer. Luciferase activity was measured by luminometer. (**a**) HIV-1 B LTR actvation by Vpr variants was comparable to wild type Vpr (**b**) HIV-1 C LTR activation by Vpr variants was almost similar to wild type. (**c**) The co-opeartive transactivation of B LTR by Vpr variants with B Tat was also comparable to B Vpr. The data shown here represents the mean value of at least three separate transfection experiments. The p value was less than 0.05 for each sample.

HIV-1 Tat plays a key role in LTR trans-activation and thus viral replication while Vpr is a weak transactivator. Therefore, the effect of naturally occurring mutations in Tat exon 1 and Vpr was investigated by comparing their ability to transactivate LTR luciferase reporter with that of wild type. The B/C recombination in Tat 71 was found to negatively affect its B LTR activation ability while B LTR trans-activation by Tat 80 was comparable to B Tat. The B LTR activation potential of other two variants was similar to that of C Tat as expected [Fig. 6(a)]. The C LTR activation by Tat 71 was found to be more than C Tat while it was almost similar to C Tat in presence of other variants [Fig. 6(b)]. Tat can also interact with Vpr and enhance the LTR activation synergistically [51]. The effect of naturally occurring mutations in Tat and Vpr on their co-operativity was also investigated. The synergistic trans-activation of B LTR by the B/C recombinant Tat 71 with B Vpr was found to be greater than than that of wild type B Tat while other Tat exon 1 variants did not show any co-operativity with B Vpr [Fig. 6(c)]. The B LTR and C LTR activation potential of Vpr variants was almost comparable to that of wild type [Fig. 7(a,b)]. The natural variations in Vpr were found to have no effect on Tat-Vpr mediated co-operative activation of B LTR [Fig. 7(c)].

### Apoptosis analysis of Tat exon 1 and Vpr variants

**Figure 8:**
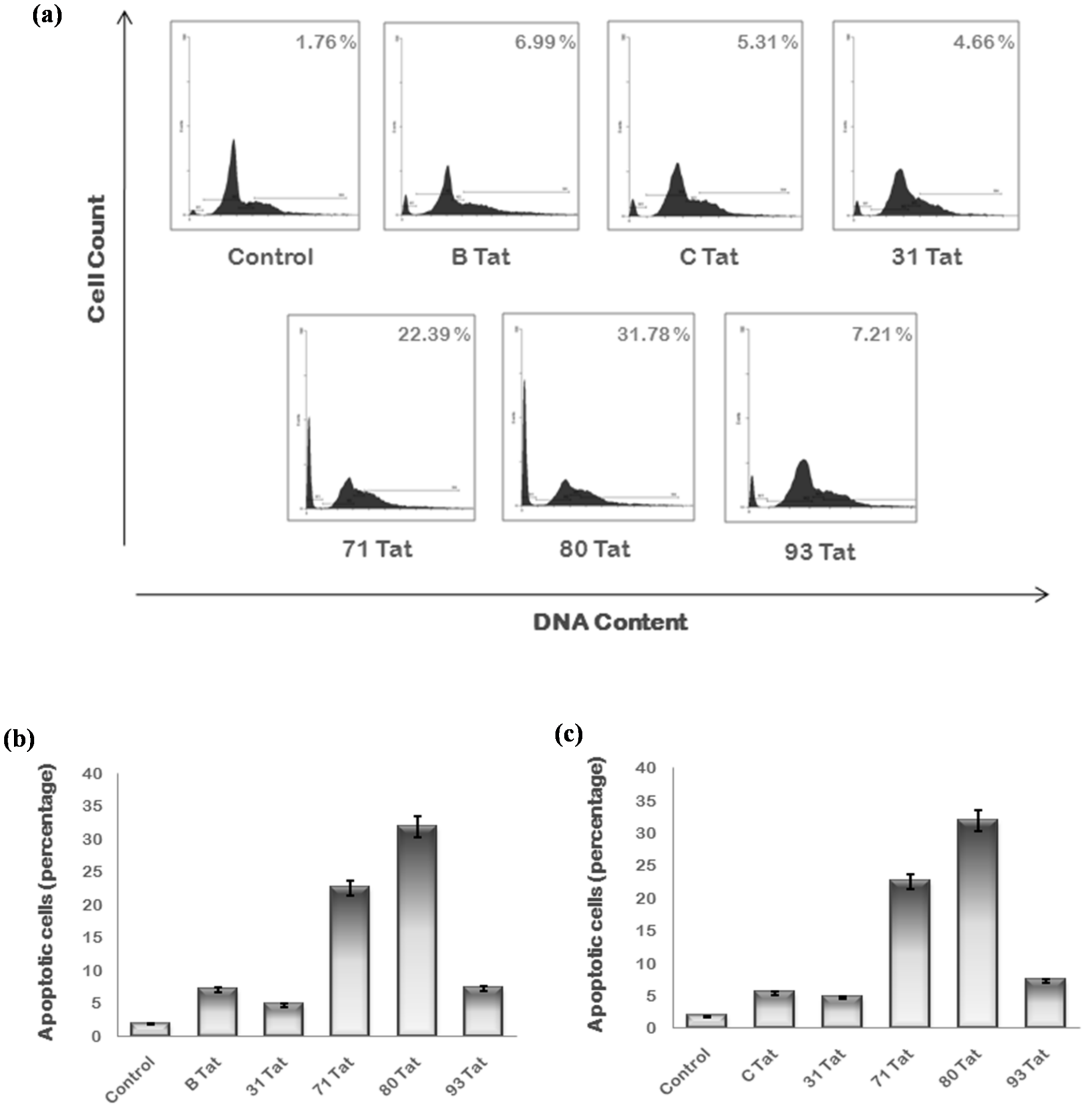
Apoptosis analysis of Tat exon 1 variants. (**a**) Hela cells were transfected with 2 µg of each plasmid encoding B Tat, C Tat and Tat exon 1 variants and cells were harvested after 24 hrs using Trypsin EDTA. Cells were then washed with PBS, fixed in 70 percent ethanol, treated with RNase A and stained with PI. After staining, cells were analyzed in PI/RNase solution by flow cytometry. Apoptotic cells were observed as a separate peak before G1 phase. Percentage apoptosis recorded with each variant is shown at the upper right corner. The B/C recombinant Tat 71 and subtype C variant Tat 80 induce more apoptosis then wild type Tat. Results shown here are the representatives of three independent experiments. (**b**) The percentage of apoptotic cells for each sample was plotted as a bar diagram comparing with wild type B Tat. The p value was less than 0.05 for each sample. (**c**) The percentage of apoptotic cells for each sample was plotted as a bar diagram comparing with wild type C Tat. The p value was less than 0.05 for each sample.

**Figure 9:**
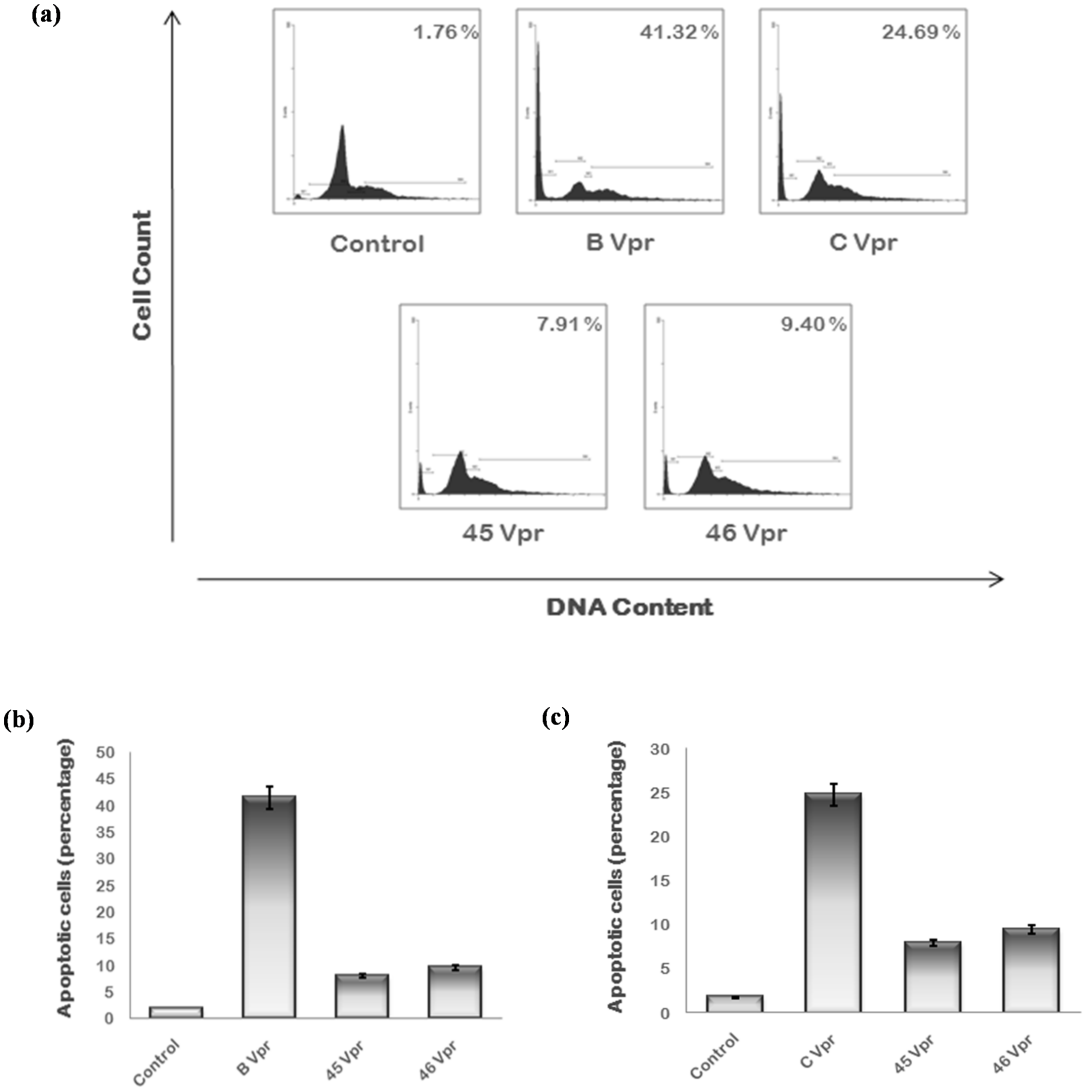
Apoptosis induced by Vpr variants. (**a**) The Hela cells were transfected with 2 µg of each plasmid encoding B Vpr, C Vpr and Vpr variants and cells were harvested after 24 hrs using Trypsin EDTA. Cells were then washed with PBS, fixed in 70 percent ethanol, treated with RNase A and stained with PI. After staining, cells were analyzed in PI/RNase solution by flow cytometry. Apoptotic cells were observed as a separate peak before G1 phase. Percentage apoptosis recorded with each variant is shown at the upper right corner. B/C/D recombinants Vpr 45 and Vpr 46 induce less apoptosis then wild type Vpr. Results shown here are the representatives of three independent experiments. **b**) The percentage of apoptotic cells for each sample was plotted as a bar diagram comparing with wild type B Vpr. The p value was less than 0.05 for each sample. (**c**) The percentage of apoptotic cells for each sample was plotted as a bar diagram comparing with wild type C Vpr. The p value was less than 0.05 for each sample.

HIV-1 Vpr and exogenously expressed Tat induce apoptosis of host cells. The apoptosis induction efficiency of Tat exon 1 and Vpr variants was measured by flow cytometry. They were transfected in Hela cells with wild type proteins as controls and after 24 hrs; cells were fixed and stained with PI. Cells were then analyzed by flow cytometer. A separate peak was observed before G1 phase representing the cells in apoptotic phase. The apoptosis induced by B/C recombinant Tat 71 (22.39%) and Tat 80 (31.78%) was much more than wild type while other variants induced apoptosis (4.66% by Tat 31 & 7.21% by Tat 93 ) to almost similar extent as that of wild type Tat [Fig. 8]. The apoptosis induction ability of Vpr variants (7.91% by Vpr 45 and 9.40% by Vpr 46) was found to be less than that of wild type (41.32% by B Vpr and 24.69% by C Vpr) [Fig. 9].

### Cell Cycle Analysis of Vpr Variants

**Figure 10:**
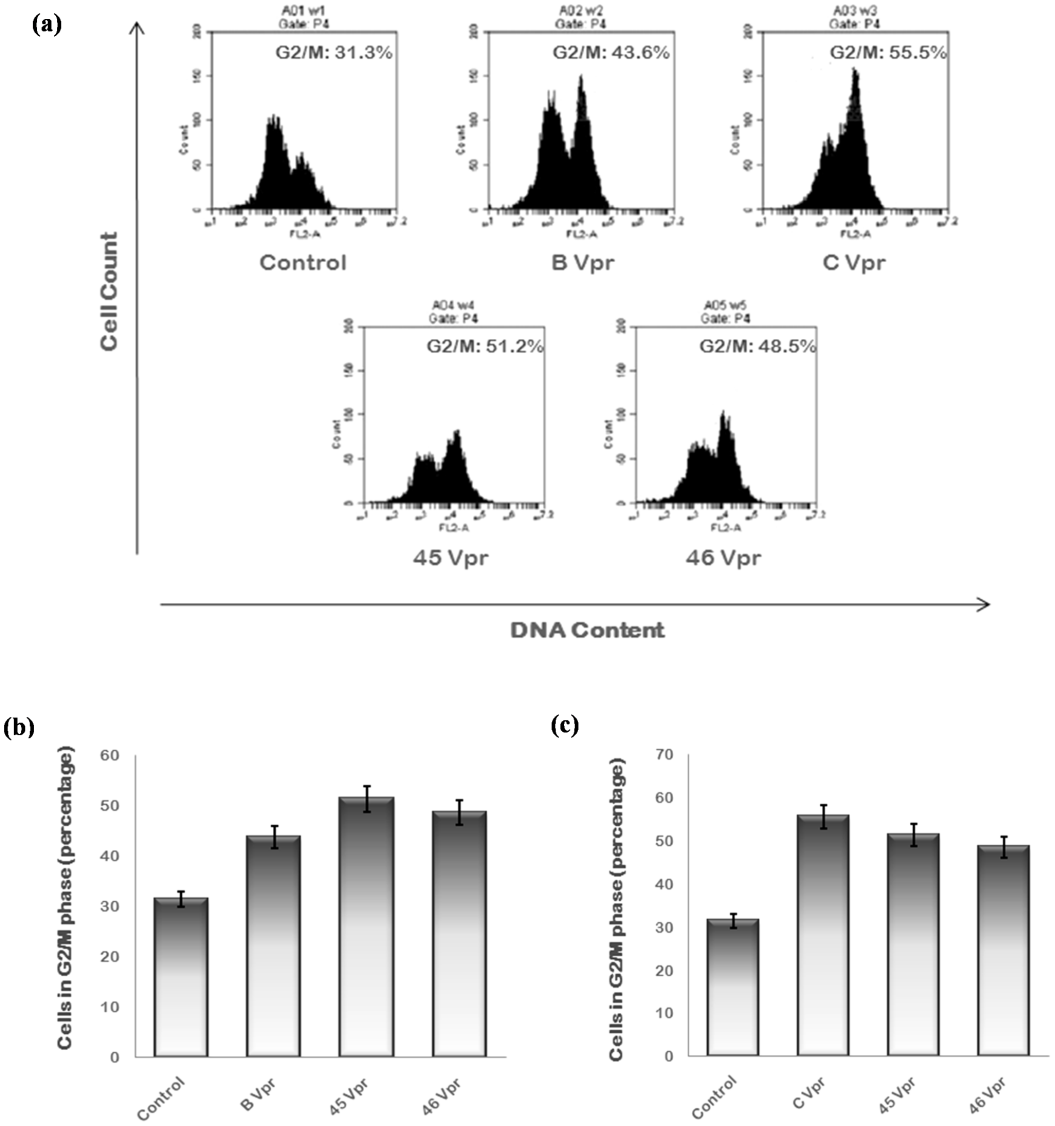
G2/M arrest induced by Vpr variants. (**a**) Hela cells were transfected with 2 µg of each plasmid encoding B Vpr, C Vpr and Vpr variants and treated with 40 µM Z-VAD-FMK (Pan caspase inhibitor). Cells were harvested after 24 hrs using Trypsin EDTA. Cells were then washed with PBS, fixed in 70% ethanol, treated with RNase A and stained with PI. After staining, cells were analyzed in PI/RNase solution by flow cytometry. The peaks of G1 - phase, S - phase and G2 - phase were obtained. Percentage of the cells in G2/M phase is mentioned at the upper right corner. Results shown here are the representatives of three independent experiments. (**b**) The percentage of cells in G2/M phase for each sample was plotted as a bar diagram comparing with wild type B Vpr. The p value was less than 0.05 for each sample. (**c**) The percentage of cells in G2/M phase for each sample was plotted as a bar diagram comparing with wild type C Vpr. The p value was less than 0.05 for each sample.

Vpr induces G2/M cell cycle arrest in infected cells. The Vpr variants were analyzed for their ability to induce G2 arrest by flow cytometry. They were transfected in Hela cells with wild type proteins as controls and cells were treated with 40 µM Z VAD-FMK [54], apoptosis inhibitor. After 24 hrs, cells were fixed, stained with PI and analyzed by flow cytometer. The G2 arrest induction potential of Vpr variants was found to be intermediate of that of wild type B Vpr and C Vpr [Fig. 10].

## Discussion

HIV-1 evolves rapidly due to its high genetic variability. The genetic analysis of natural variants of *tat* exon 1 and *vpr* revealed interesting features. Most of the amino acid variations in Tat were found in cystein rich and core regions while N-terminal region, TAR binding region and glutamine rich region (except one mutation) were almost conserved in all sequences. One variant, Tat 71, was found to be a recombinant of subtype B and C with three point mutations (K29R, I39M and Y47H) while others resembled subtype C with some point mutations (F38L, L43V, S46F, K29R, C30R, Y47H and I39M). Tat 71 sequence was also analyzed by Sim Plot analysis which showed that it is a B/C recombinant with one point of recombination between subtype B and C. Three Vpr variants were mosaic of subtype B, C and D with one point mutation (M59T) and different from B/C/D recombinant of HIV-1 Vpr already reported [58]. Vpr 79 showed premature stop codon formation. Although sequence of Tat exon 1 variants was similar to C Tat with some point mutations (except Tat 71), their stability and expression was similar to that of wild type B Tat. This may be due to the mutations naturally present in their sequence. There is no previous report showing the effect of these naturally occurring mutations on expression and stability.

Functionally, Tat exon 1 variants were found to have varying LTR activation potential. There was almost no effect of reported B/C/D recombination on LTR activation ability of Vpr variants while the previously reported B/C/D recombinant of Vpr [58] was more active than wild type. The activation of HIV-1 subtype B LTR by Tat 80, which resembled subtype C except two point mutations (L43V and S46F), was more than wild type C Tat. However it is reported that Tat is phosphorylated at S16 and S46 which is required for LTR directed transcription of HIV-1 genes and mutation S16 and S46 residues results in reduced phosphorylation of Tat and hence inhibited HIV-1 replication [59] but this mutation (S46F) of a hydrophilic amino acid (S) to a hydrophobic amino acid (F) at position 46 may enhance the hydrophobic property of the Tat core region [60] which is essential for binding to Cyclin T1 [61] necessary for Tat function [18], [62]. Also, substitution of aromatic with non-aromatic amino acids leads to 2-fold reduction of Tat activity [63]. So, substitution of non-aromatic amino acid (S) by aromatic amino acid (F) at position 46 may increase the activity of Tat. The B LTR activation by Tat 31 and Tat 93 was almost similar to that of C Tat. Both Tat 80 and Tat 93 have two point mutations which were same (L43V and S46F) but B LTR transcriptional activity of Tat 93 is relatively less than that of Tat 80. It may be due to the mutation of C30. This mutation has been found to decrease the trans-activation potential of Tat [64], [17], [65]. The B/C recombination with three point mutations (K29R, I39M and Y47H) in Tat 71 reduced its B LTR trans-activation ability. It is reported that the amino acid residues at the positions 35 and 39 in Tat are strongly co-related and mutation of either residue of this pair of amino acids results in a Tat mutant that fails to activate the viral LTR. However, simultaneous introduction of both mutations restores gene function to wild-type [66]. But Tat 71 having I39M mutation is still able to activate B LTR transcription. This may be due to the other mutation Y47H which is reported to revert the loss of activity of Y26A Tat mutant despite the fact that both of these tyrosines are important for HIV-1 replication [63] and Y47H mutation, when introduced as an individual mutation in wild type Tat, increases the activity of mutant Tat two fold in transient assays [67]. K29R mutation was also observed in Tat second site revertants but it was not found to improve the activity of Tat in LTR transcription assays [67].

HIV-1 subtype C LTR activation by Tat 71 was more than that of C Tat and comparable to B Tat which may be due to B/C recombination. The C LTR transcriptional activity of Tat 80 was found to be more than C Tat. This may be due to the presence of S46F mutation in its sequence [60]. The C LTR transcription was comparable to C Tat in case of Tat 31and Tat 93. The C LTR transcriptional activity of Tat 93 is relatively less than that of Tat 80 despite same point mutations at two sites (L43V and S46F) due to the reason described above for B LTR activation.

Although, Tat 71 had reduced B LTR transctivation, it showed more co-operativity with B Vpr in B LTR activation than wild type B Tat. So, it may be concluded that Tat 71 sequence will lead to enhanced replication in viral context and thus positively selected in evolution. Other three variants did not show any co-operative activation of B LTR with B Vpr as expected because their sequence resembled that of C Tat except few point mutations. There was also no effect of naturally occurring variations in Vpr on its co-operativity with Tat. It suggested that Vpr only plays second fiddle to the LTR transactivation function of Tat.

It may be argued that the functional differences in samples could be because of the changes in their corresponding LTR sequences. Therefore, we amplified LTR sequences from two representative samples (Tat 71 and Tat 80) using the protocol as described by us earlier [68]. When compared to consensus LTR sequences, the sample LTR sequences showed conservation of all the major transcription factor binding sites (TATA box, Sp1, NF-kB, NFAT-III, AP-1, AP-2) (data not shown). Thus, we concluded that the observed functional differences are due to the changes in Tat sequence itself.

The variations found naturally in Tat exon 1 and Vpr were also found to affect their apoptosis induction ability. The apoptosis induced by Tat 31 and Tat 93 was almost similar to wild type Tat while it was much more than B and C Tat in case of Tat 71(B/C recombinant) and Tat 80. Thus, the mutations, which enhanced the transcriptional activity of Tat, also increased the apoptosis induction potential of Tat. Both Vpr variants induced less apoptosis than wild type but normal G2 arrest. Thus B/C/D recombination found in Vpr variants had negative impact on their apoptosis but not G2 arrest induction potential.

Overall, four point mutations occurring naturally in Tat exon 1 were found to be functionally significant. S46F and Y47H mutations were increasing the activity of Tat while C30R and I39M were negatively affecting Tat activity. Other mutations (K29R, L43V and F38L) occurring naturally in Tat exon 1 were found to be functionally inactive. The B/C/D recombination in Vpr variants was found to negatively affect their apoptosis induction capability, the characteristic function of Vpr but no effect on other functions. Thus, these variations including inter-subtype recombination occurring naturally in HIV-1 proteins have a strong impact on the pathogenesis of virus. Several studies have indicated that, recombination may take place under the selective pressure imposed by antiretroviral drugs or due to co-infection with different strains resulting in new HIV-1 variants with dual drug resistance, altered tissue tropism, pathogenicity, and host range, or with changes in antigenic epitopes [69], [70], [71]. No effective vaccine against HIV is available till date despite several efforts using HIV-1 proteins. Perhaps the type and spread of variations in the proteins of infecting strains would impact efforts for a purposive vaccine. The genetic and functional analysis of HIV-1 gene variants circulating in population will furnish the knowledge to understand the HIV-1 biogenesis and its evolutionary process. This will be useful for designing new anti-HIV therapies and vaccine strategies in the future.

## Supporting Information

Figure S1 **Boot scan analysis of non-recombinant Tat exon 1 variants.** Sequences of Tat 31, Tat 80 and Tat 93 were analyzed by Sim Plot. They were aligned with HIV-1 subtype B, C and D consensus sequences and subjected to the boot scan analysis. All of them were similar to C Tat. (**a**) Boot scan analysis of Tat 31 (**b**) Boot scan analysis of Tat 80 (**c**) Boot scan analysis of Tat 93.
(TIF)
