# Supplementary figures and images for: Effect on HIV-1 Gene Expression, Tat-Vpr Interaction and Cell Apoptosis by Natural Variants of HIV-1 Tat Exon 1 and Vpr from Northern India
Source: PLoS One. 2013 Dec 19;8(12):e82128. doi: 10.1371/journal.pone.0082128 (PMC3868622; doi:10.1371/journal.pone.0082128)

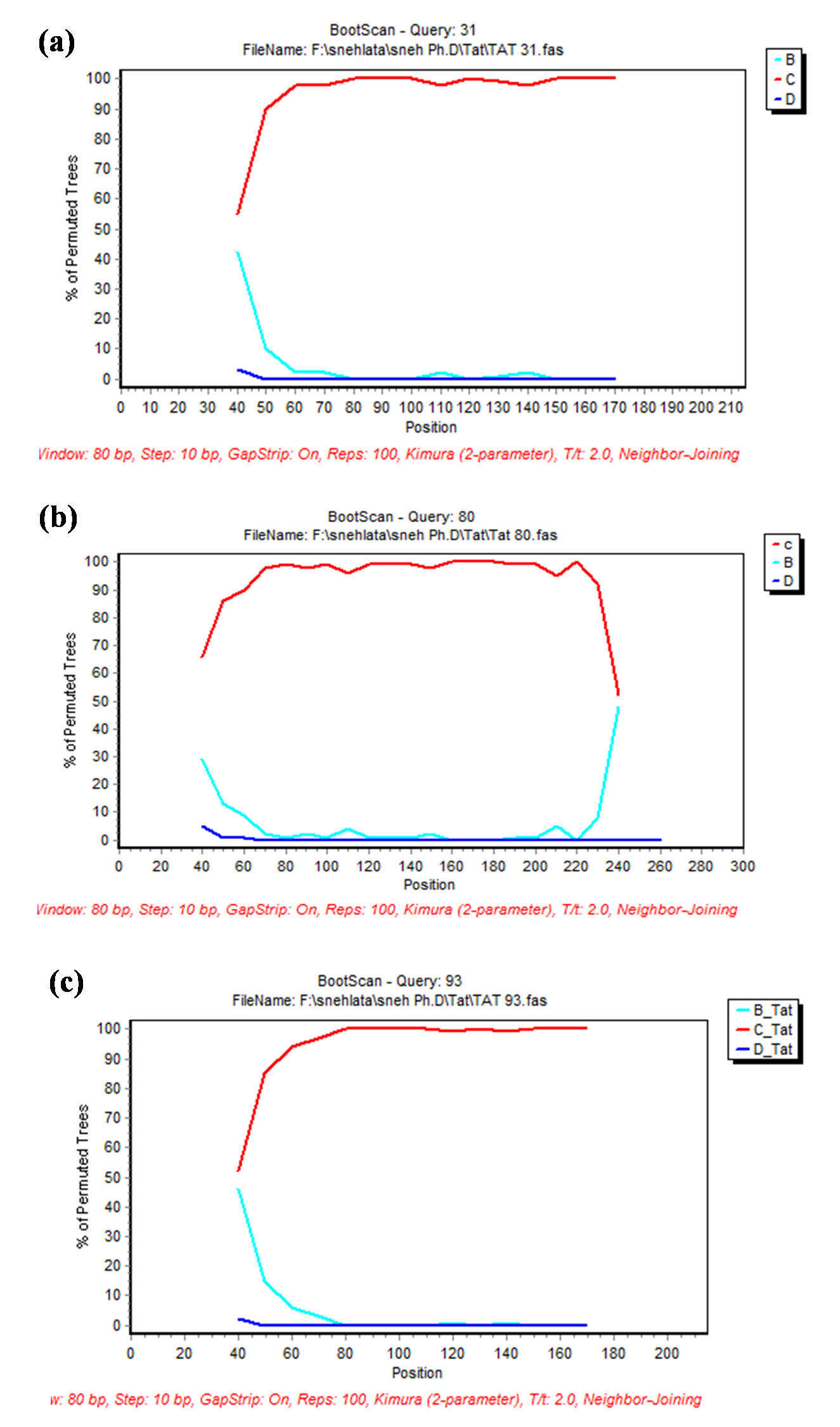

Supplement: Figure S1 — Boot scan analysis of non-recombinant Tat exon 1 variants. Sequences of Tat 31, Tat 80 and Tat 93 were analyzed by Sim Plot. They were aligned with HIV-1 subtype B, C and D consensus sequences and subjected to the boot scan analysis. All of them were similar to C Tat. (a) Boot scan analysis of Tat 31 (b) Boot scan analysis of Tat 80 (c) Boot scan analysis of Tat 93. (TIF) [file pone.0082128.s001.tif]
